# Can Chemotherapy be Integrated with Brachytherapy in Locally Advanced Carcinoma Cervix- A Proof of Principle Study

**DOI:** 10.31557/APJCP.2019.20.9.2653

**Published:** 2019

**Authors:** Subhash Gupta, Prashanth Giridhar, Dayanand Sharma, Haresh K P, Julka P K, Goura K Rath

**Affiliations:** *Department of Radiation Oncology, All India Institute of Medical Sciences, New Delhi, India.*

**Keywords:** Carcinoma cervix, high dose rate brachytherapy, chemo, brachytherapy

## Abstract

**Introduction:**

Cancer of the cervix is the second most common cancer in women in India. Chemoradiotherapy is the standard treatment for locally advanced carcinoma cervix. Chemotherapy is not given on days of brachytherapy due to the fear of increased toxicity though studies supporting or refuting it are limited. We intended to study feasibility of adding chemotherapy to brachytherapy with assessment of acute toxicity and response rates.

**Methods:**

29 patients of locally advanced carcinoma cervix (FIGO IIB to IIIB) were assigned to receive either three sessions of high dose rate (HDR) brachytherapy alone or HDR brachytherapy with concurrent chemotherapy of Paclitaxel and Carboplatin after completion of external beam radiation with concurrent Cisplatin. Patients were assessed for compliance of treatment, toxicity and response rates at three and six months. The p-value less than 0.05 was considered statistically significant. Fischer’s exact test was used for statistical analysis.

**Results:**

15 patients were assigned to the standard of care arm and 14 patients to the experimental chemo-brachytherapy arm. The median number of cycles of chemotherapy possible with brachytherapy was two (Range: 1 -3). At three months after treatment all patients except one patient in each arm had a complete response. There was two acute grade 3 hematological toxicity and two acute grade 3 or higher gastrointestinal toxicity in the experimental arm but none in the standard arm. The experimental arm had a statistically higher incidence of acute grade 3 and 4 toxicity than the standard arm (p=0.042).

**Conclusions:**

Chemo-brachytherapy is associated with higher acute toxicity with comparable response rates. Small patient numbers and short follow up impedes us from providing conclusive evidence.

## Introduction

Cervical cancer is the second most common cancer among women in India (Globocan, 2018). 96,222 new cases of cervical cancer are diagnosed every year in India with over 60,000 deaths every year due to cervical cancer (Globocan, 2018). The 5 year survival for FIGO stage II disease ranges from 50-70% and that of stage III from 30-50% when treated with standard of care chemoradiotherapy. Chemoradiotherapy of carcinoma cervix includes both external beam radiation (EBRT) and brachytherapy. In practice, chemotherapy is integrated with radiation only during the pelvic EBRT phase and not integrated with brachytherapy due to anticipated toxicity though studies supporting or refuting this approach are limited. Various dose fractionations have been used in brachytherapy for carcinoma cervix with dose per fraction of high dose rate (HDR) brachytherapy ranging from 5.5 Gy – 9 Gy (Viswanathan 2012; Ozsaran et al., 2003;Sood et al., 2003). HDR brachytherapy of 9 Gy per session for two or more sessions has been reported to be well tolerated without increased toxicity (Ozsaran et al., 2003; Sood et al., 2003). We therefore, hypothesized HDR Brachytherapy of 7 Gy per fraction with concurrent chemotherapy will be well tolerated without increased toxicity. The addition of chemotherapy agent Paclitaxel may give added benefit by re-assorting cancer cells into a more radiosensitive G2M phase. Carboplatin induces radio-sensitization similar to Cisplatin with decreased risk of nephrotoxicity. The primary objective of this study was to compare the toxicity profile and compliance between HDR brachytherapy alone and HDR brachytherapy with concurrent paclitaxel and carboplatin in locally advanced carcinoma cervix. The clinical response rates and disease free survival at 3 and 6 months were also analyzed in both the study arms. 

## Materials and Methods

Patients of 20 - 65 years of age with diagnosis of squamous cell carcinoma of cervix, FIGO stage IIB - IVA, Karnofsky performance status of 70 or higher, normal complete blood counts, liver and renal function tests were considered eligible. Patients with uncontrolled co-morbid condition(s) of hypertension, diabetes mellitus, coronary artery disease or chronic renal disease, abnormal blood counts, liver and renal function parameters precluding chemotherapy, pregnancy, lactation or history of prior malignancy were excluded. Patients who received less than 4 cycles of concurrent chemotherapy with EBRT were also excluded. Baseline FIGO stage before EBRT was noted based on initial clinical findings and computed tomography imaging of abdomen and pelvis. The patients were assigned continuously one after another between the treatment and experimental arms after completion of EBRT. External Beam Radiotherapy (EBRT) was delivered to a dose of 50.4 Gy in 28 fractions over 5.5 weeks with weekly Cisplatin at a dose of 40 mg/m2 by four field box technique in Medical Linear accelerator with 6/15 Mega voltage photon energy. External beam radiation planning was done by conventional 2-D simulation with a simulator with a four field box technique. The lateral borders were kept 1.5 cm across the widest point of pelvic brim and upper border placed at L4-L5 junction. The anterior border in the lateral field was placed at the anterior border of pubic symphysis and posterior border at S2-S3 junction. If vagina was involved the entire vagina was treated with a marker placed at the introitus. If not involved, the fiducial was placed at the lowest point of growth and lower border kept 3 cm below growth. 

**Table 1:** Patient Characteristics and Treatment Outcomes

	Experimental arm (n=14)	Standard of care arm (n = 15)
Median Age	52.5 years	45 years
Stage		
IIIB	6 (43 %)	10 (66 %)
IIIA	1(7 %)	0
IIB	7 (50 %)	5 (34 %)
Hb		
Median	10.1gm/dl	10.9 gm/dl
<10 gm/dl	5 (36 %)	3 (20 %)
>10 gm/dl	9 (64 %)	12 (80 %)
Concurrent Chemotherapy (EBRT)		
Median	4.5	5
Range	3-6	3-6
Concurrent Chemotherapy (BT)		
Median	2	NA
Range	1-3	
Response After EBRT		
CR	4	5
PR	9	10
SD	1	0
PD	0	0
Response after BT (3 months)		
CR	13	14
PR	1	0
PD	0	1 (Para aortic Node)
Status at last FU		
CR	13	14
PD	0	1
Grade I /II toxicity (with ICRT)		
Haematological	3	2
GI	2	1
Grade III and IV toxicity (with ICRT )		
Haematological	2	0
GI	2	0
*Statistical Analysis of Gd Iii or Iv Toxicity		(p value = 0.042)

**Figure 1:**
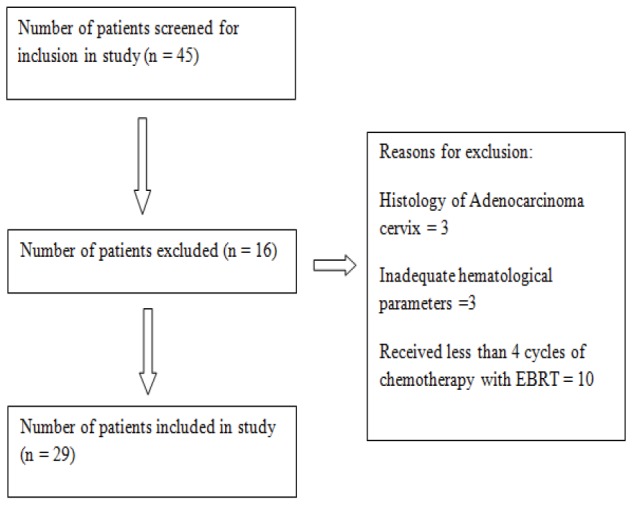
Consort Diagram of Patient Selection

Patients were considered for Intracavitary radiotherapy (ICRT) as early as possible after completion of EBRT (preferable within one week of EBRT). ICRT application was done using modified Fletcher-Suit applicator in an operation theater under sedation and analgesia with Promethazine and Pentazocine. After the procedure a planning CT scan was acquired in the Philips Brilliance big bore CT scanner. The treatment plan was generated in Oncentra treatment planning system and treatment was delivered in HDR micro-selectron v-3 machine. A dose of 7 Gy to point A was prescribed in each session. Three sessions of ICRT was performed for each patient, one week apart. In the experimental arm patients were administered Inj. Paclitaxel (40 mg/m^2^) and Inj. Carboplatin (AUC2) over a period of two hours, four hours before insertion of the applicator. During treatment, patients were monitored for any toxicity and were graded as per CTCAE v 4.03. The complete blood counts were checked every week during EBRT and before each session of ICRT. After completion of ICRT, complete blood count was done one month later and then at three and six months. The patients were monitored with history and clinical examination during treatment to look for toxicity.

Chemotherapy was stopped on occurrence of grade 3 or higher hematological / gastrointestinal toxicity. After completion of brachytherapy/chemo-brachytherapy, the patients were followed up at 1, 3 and 6 months. The patients were evaluated clinically and contrast enhanced computed tomography as required. Response assessment was done at three and six months after ICRT with clinical examination and at three months after completion of ICRT with contrast enhanced CT imaging of abdomen and pelvis and chest X ray. The responses were graded then as complete response, partial response, stable disease or progressive disease as per RECIST v1.1. The study was approved by the institutional review board and ethical committee. 

*Statistical analysis*

Data was recorded on a pre-designed proforma and entered on an Excel spread sheet. Categorical variables were summarized by frequency (%) and quantitative variables were summarized by median and range. The p-value of less than 0.05 was considered statistically significant. Fischer’s exact test was used for statistical analysis on STATA version 12 software.

## Results

*Patient characteristics*

A total of 45 patients were screened for inclusion in this protocol. 29 patients fulfilled the inclusion criteria (Figure 1). A total of 15 patients were accrued in the standard of care arm and 14 patients in the experimental arm.The median age of patients in experimental arm was 52.5 years (Range 37 – 65 years) and control arm was 45 years (Range 35 – 65 years). No patient had family history of cancers, documented pelvic inflammatory diseases, sexually transmitted diseases or HIV infection. All patients in the experimental arm and 14 of 15 patients in the control arm had history of multiple pregnancies. Patient characteristics have been summarized in Table 1.

Treatment characteristics: Median number of chemotherapy cycles with EBRT was 4.5 (Range: 3 – 6) and 5 (Range: 3 – 6) in the experimental and standard of care arm respectively. Median number of chemotherapy cycles with brachytherapy was two (Range: 1 – 3).The treatment details are summarized in Table 1.

Clinical response and statistical analysis: At six months after completion of treatment, 13 of 14 patients in the experimental arm and 14 of 15 patients in the standard of care arm had a complete response. One patient in the experimental arm had partial response and one patient in the standard of care arm developed progressive disease (para-aortic nodal recurrence). The median follow up of patients was 6.5 months (Range: 6 months – 9.5 months). Given the limitation of inadequate events, planned disease free survival analysis could not be done.

*Toxicity and statistical analysis*

In the experimental arm, three patients had hematological and five patients had documented lower gastro-intestinal toxicity during external beam radiation therapy. In the standard of care arm, two patients had hematological toxicity and one patient had grade 2 lower gastro-intestinal toxicity during external beam radiation therapy. In the experimental arm, following chemo-brachytherapy two patients developed grade 3 anemia, one patient developed grade 2 anemia and two patients developed grade 1 thrombocytopenia. Chemotherapy was avoided with brachytherapy after patients developed grade 3 anemia. In the experimental arm, one patient developed grade 3 and one patient developed grade 4 lower gastro-intestinal toxicity. The patient with grade 4 toxicity presented to the emergency department two days after first session of chemo-brachytherapy with history of diarrhea in hypovolemic shock with hyponatremia. The patient was managed with fluids and inotropic support. The patient was salvaged successfully and completed two sessions of brachytherapy two weeks later.In the standard of care arm, no grade 3 or 4 side effects were seen. Statistical analysis of occurrence of grade 3 or 4 toxicity between treatment arms resulted in a significantly higher toxicity in the experimental arm (p= 0.042).

Toxicity on follow up: During follow up, one patient in the experimental arm developed peripheral neuropathy and nail dystrophy and two patients developed lower backache radiating to thighs. No history of trauma could be elucidated. Contrast enhanced computed tomography revealed no evidence of disease or sacral insufficiency fractures. Vaginal stenosis developed in two patients in experimental arm and two patients in the standard of care arm. In the experimental arm, one patient complained of bleeding per rectum at last follow up 9 months post treatment. A proctoscopy revealed two punctate hemorrhagic spots in the anterior rectal wall suggestive of radiation proctitis. One patient in the standard of care arm developed hematuria 9 months post treatment. Cystoscopy revealed no specific bleeding spots but a few raw areas were seen. 

## Discussion

Radiotherapy is the mainstay of treatment for locally advanced carcinoma of the cervix. In 1999, five large randomized controlled trials demonstrated the efficacy of chemoradiotherapy using CDDP (cisplatin) based regimen (Tseng et al., 1997; Colombo et al., 1997; Whitney et al., 1999; Rose et al., 1999; Keys et al., 1999; Morris et al., 1999; Peters et al., 2000;). Subsequently, meta-analyses also showed improved overall survival in patients treated with CCRT (Green et al., 2001; Lukka et al., 2002). CCRT followed by brachytherapy thus was established as the standard treatment for locally advanced carcinoma cervix. High dose rate (HDR) and low dose rate (LDR) brachytherapy seem to be relatively equivalent treatments in terms of survival outcomes based on existing retrospective and prospective studies (Stewart et al., 2006; Patel et al., 1994; Fu et al., 1990; Hareyama et al., 2002; MInoue et al., 2003; T Teshima et al., 1993; TWang et al., 2010; Petereit et al., 1999; Lertsanguansinchai et al., 2004;).

With present treatment modalities, the 5 year survival is 30 – 50 % for locally advanced carcinoma of the cervix. Patients fail both locally and at distant sites like para-aortic nodes and liver. The reason could be the presence of microscopic residual disease even after apparent complete clinical response. Other reasons could be inadequate target coverage of the primary during brachytherapy in the setting of CT-simulation image guidance alone with point A prescription. Steps need to be taken to circumvent these problems. Image based brachytherapy is being followed in many centers to improve target coverage. The logical step to circumvent the problem of microscopic residual disease would be to further integrate chemotherapy to take care of systemic micro-metastatic disease. Integration of chemotherapy with brachytherapy may hypothetically have radiobiological benefits as well. Paclitaxel has been shown to re-assort cells to a more radiosensitive G2M phase of cell cycle. Carboplatin provides radio-sensitization similar to Cisplatin with decreased risk of cumulative nephrotoxicity. The dose of concurrent chemotherapy in our study was decided based on a phase 1 dose finding study (Rao et al., 2005; ). The maximum tolerated doses for a phase II trial were found to be 50 mg/m2 and AUC 2.5 for Paclitaxel and Carboplatin respectively with external beam radiation. A slightly lower dose was chosen keeping in mind the general condition of patients in the region. Our study is a pilot study to test primarily the feasibility and toxicity profile of such an approach.

In our study, patients in the experimental arm did develop clinically significant hematological (n = 2) and lower gastro-intestinal toxicity (n = 2) which was statistically significant (p = 0.042) when grade 3 and 4 hematological and gastro-intestinal toxicity were analyzed together. Our study does have a few limitations. First, the sample size is too small to provide significant power to the study. Second, there was no randomization done in the study. This introduces bias into the study. Third, chemotherapy with brachytherapy was deferred on appearance of grade 3 or higher side effects. So, the full toxicity due to integration of chemotherapy is probably under reported. Fourth, the follow up period is too short to assess late effects of radiation. All patients received radiation to the pelvis with chemotherapy before being assigned to treatment arms. This would have led to bone marrow suppression. This could have led to higher or lower occurrence of hematological toxicity in the experimental versus control arm. In the INTERTECC 2 phase 2 trial, the role of IMRT in reducing hematological and gastrointestinal toxicity in locally advanced carcinoma cervix was evaluated. A pre-planned sub-group analysis of role of PET guided IMRT in reducing acute neutropenia was done. IMRT reduced acute gastrointestinal and hematological toxicity and PET guided IMRT reduced acute neutropenia in the study (Mell et al., 2017;). So, IMRT planning for EBRT could help decrease toxicity further. A well-designed randomized phase III randomized controlled trial of concurrent chemo-brachytherapy with longer follow up is warranted to look into the impact of this approach in locally advanced carcinoma cervix.

In conclusion, chemo-brachytherapy seems feasible in locally advanced carcinoma of the cervix though higher hematological and gastrointestinal toxicity was documented in the chemo-brachytherapy arm. We would like to emphasize necessity of exploring this hypothesis in a well-designed phase II randomized controlled trial with larger sample size and longer follow up.
